# DMNet: A Frequency-Enhanced and Adaptive Spatial Fusion Network for RGB–Infrared Object Detection

**DOI:** 10.3390/s26123625

**Published:** 2026-06-06

**Authors:** Yuchen Yao, Xinlong Wang, Yan Liu

**Affiliations:** 1 Hubei Key Laboratory of Petroleum Geochemistry and Environment, Yangtze University, Wuhan 430100, China; yuchen_yao.stu@yangtzeu.edu.cn (Y.Y.);; 2 College of Resources and Environment, Yangtze University, Wuhan 430100, China

**Keywords:** RGB–infrared fusion, multimodal object detection, frequency-domain features, small object detection, adaptive fusion

## Abstract

Object detection in complex environments remains challenging due to illumination variations, background clutter, and the presence of small objects. Multimodal detection methods based on RGB and infrared (IR) data have shown promising potential by leveraging complementary information across modalities. However, existing approaches still suffer from cross-modal feature misalignment, loss of fine-grained details, and insufficient semantic interaction. In this work, we introduce a novel dual-stream framework called DMNet, specifically tailored for visible and IR multimodal object detection. The architecture integrates four core components designed to tackle these challenges: surface detail fusion (SDF) for shallow feature alignment, wavelet feature extraction (WFE) for frequency-domain enhancement, context-guided enhancement (CGE) for semantic refinement, and adaptive spatial fusion (ASF) for multi-scale feature aggregation. We conduct extensive evaluations on three benchmark datasets, including M3FD, LLVIP, and VEDAI, demonstrating that DMNet achieves superior detection performance compared with existing methods. Experimental results confirm that DMNet outperforms existing approaches, achieving an mAP@0.5 of 78.4% on M3FD, 94.4% on LLVIP, and 59.0% on VEDAI. Notably, the model maintains a relatively compact parameter scale (5.72 million parameters) while achieving superior detection performance, making it suitable for practical deployment. These findings highlight DMNet as an effective and efficient solution for multimodal object detection under challenging conditions, especially in low-light and small-object scenarios.

## 1. Introduction

### 1.1. Background and Significance

As intelligent sensing and computer vision continue to evolve rapidly, object detection has become a fundamental task in numerous applications, including autonomous driving, intelligent surveillance, and unmanned systems [1]. In recent years, deep learning-based detectors, including Faster R-CNN [2], YOLO series [3], and Transformer-based models [4], have significantly improved detection accuracy and efficiency.

However, most existing detection frameworks are designed for single-modality input, which limits their robustness under adverse scenarios like poor lighting, object blockage, and cluttered surroundings. To overcome these limitations, multispectral object detection has been widely studied by integrating RGB and IR modalities [5]. RGB offers detailed texture and hue information, while IR is insensitive to illumination variations and can effectively highlight thermal targets in low-light environments [6].

Recent studies have demonstrated that the fusion of RGB and IR information can significantly enhance detection performance in challenging environments [7]. Furthermore, multispectral perception has shown great potential in improving small object detection and nighttime detection tasks [8].

Therefore, the development of efficient and robust RGB–IR dual-modality detection frameworks has become a key research focus in intelligent sensing and multimodal perception.

Although existing multispectral object detection methods have achieved considerable progress in specific scenarios, several fundamental limitations remain. In particular, these approaches often struggle to simultaneously model holistic contextual understanding and retain fine-grained spatial details of dual-modality features, resulting in limited robustness under complex backgrounds and illumination variations. Further, inaccurate cross-modal alignment further leads to increased bounding box localization errors, and the missed detection of dense or small-scale targets remains a prominent issue. In addition, many existing models also lack efficiency, making them difficult to deploy in real-time or resource-limited settings. The discriminability of fused features is often insufficient due to ineffective interaction between modalities, especially when managing multi-scale targets that exhibit significant scale variation. Consequently, conflicts across feature scales are difficult to resolve, severely limiting the small object detection and overall system performance.

### 1.2. Main Contributions

Aiming at the challenges present in existing RGB-IR/thermal imaging dual-mode object detection methods, including cross-modal feature fusion detail loss, misalignment in shallow-level structural correspondence, and noise interference in high-level fusion, we present DMNet, a multimodal detection framework composed of several carefully designed modules, including SDF, WFE, CGE, and ASF. The main contributions are summarized as follows:

(1) SDF: To address the issues of detail redundancy, feature information loss, and inaccurate shallow structure alignment that often occur in traditional pixel-level fusion in multimodal detection, we introduce SDF (surface detail fusion). This module introduces a channel–spatial dual attention mechanism at the shallow layer of the feature extraction network. By concatenating the IR and visible light features and combining global average pooling and point-wise convolution to generate fusion weights, it achieves adaptive enhancement and dynamic fusion of cross-modal features, thereby suppressing background noise while retaining key details, and significantly improving the low-level feature expression ability.
(2) WFE: To expand the receptive field while avoiding the computational overhead caused by large convolution kernels, we introduce WFE (wavelet feature extraction) in the backbone network. This structure uses wavelet transformation to decompose the features into different frequency sub-bands, strengthening low-frequency structural features while preserving high-frequency details, enabling the model to obtain a larger receptive field with lower parameters, thereby improving the feature expression ability for complex scenes and multi-scale targets.
(3) CGE: To improve the model’s capability for representing target semantics under complex backgrounds, we introduce CGE (context-guided enhancement) at the network neck. This module jointly extracts local features, surrounding context information, and global semantic information, and combines the residual connection mechanism to achieve feature enhancement, effectively improving the feature expression ability, which helps it handle complex scenes and multi-scale targets in challenging environments more effectively.
(4) ASF: To mitigate conflicts across multi-scale features within the feature pyramid, we design the ASF (adaptive spatial fusion) head mechanism in the detection head. This module learns the spatial weights of different hierarchical features to perform adaptive multi-scale feature integration, enabling the network to automatically select the most effective feature information, thus improving the detection performance for small and occluded targets.

The remainder of the paper is organized as follows: Section 2 introduces related research works and reviews the current visible light and IR multimodal target detection methods; Section 3 details the network structure and key module design reported in this paper, including SDF, WFE, CGE, and ASF, etc. as the core module designs; Section 4 presents the experimental settings and evaluation metrics, and conducts ablation experiments and comparative experiments analysis on multiple datasets; Section 5 further verifies the model’s generalization performance through cross-dataset experiments; finally, Section 6 concludes the paper and discusses future research directions.

## 2. Related Works

### 2.1. Single-Modality Object Detection

Early deep learning-based detectors were divided into two-stage and one-stage categories. Two-stage methods like R-CNN [1], Fast R-CNN [9], and Faster R-CNN [10] first propose candidate regions, and then classify and regress them, which have high accuracy but large computational costs; one-stage approaches such as the YOLO series [1,11,12,13,14] and SSD [15] directly predict bounding boxes and categories, balancing speed and accuracy. Additionally, detector models like DETR [4], RT-DETR [16], and DINO [17] introduce global self-attention mechanisms to achieve end-to-end detection, but they have slow convergence and high computational costs.

In single-modal imaging, RGB images are rich in texture and color information when the lighting is good, which is conducive to fine recognition, but their performance significantly declines in conditions such as at night, in haze, smoke, or with occlusions [5,18]; IR imaging relies on thermal radiation and can still maintain the target outline in low light or adverse weather conditions, compensating for the shortcomings of RGB [5,18]. Experiments show that on datasets such as FLIR, LLVIP, and VEDAI, single-modal IR detection typically has a higher mAP than RGB; for example, the IR mode of YOLOv5 on LLVIP has an mAP@50 of 96.6%, significantly higher than the 90.8% of the RGB mode [5,19], indicating that IR has stronger recognition ability in adverse conditions such as low light, but single-modal methods lack cross-modal complementarity and have difficulty coping with complex environments in all weather conditions.

### 2.2. Multispectral Object Detection

Multispectral (RGB+IR) object detection improves performance under complex environments by leveraging complementary information across modalities. Hwang et al. [5] established an early RGB-T pedestrian detection benchmark, where hand-crafted feature aggregation (ACF) was adopted to achieve cross-modal detection, thereby initiating research on feature-level fusion. Subsequent studies compared three fusion strategies, including image-level, feature-level, and decision-level fusion [20], and demonstrated that feature-level fusion achieves better performance by preserving modality-specific characteristics [21].

In deep learning-based approaches, CFT (Cross-Modal Fusion Transformer) utilizes self-attention to capture distant cross-modal dependencies, thereby enhancing the discriminability of fused features [19]. TSRA (Translation–Scale–Rotation Alignment) predicts translation, scale, and rotation offsets to alleviate weak alignment issues and is further integrated with CFT to form the CAGT detection head, achieving improved results on the DroneVehicle and KAIST datasets [10,12,13,14].

Furthermore, the EFETN framework adopts a dual-stream Transformer backbone. Prior to fusion, a detail enhancement module (DEM) is employed to extract and enhance edge and contextual information from RGB and IR modalities, respectively. Subsequently, a cross-modal fusion Transformer module (CFTM) is proposed to enable mutual enhancement between modalities, delivering top-tier results on FLIR, LLVIP, and VEDAI datasets [5,19,20,22,23,24,25].

Lightweight design has also become an important research direction. FQDNet enhances feature representation by introducing a channel shuffle-based downsampling block (CSSB) and a spatial-channel attention fusion module (SCAFM), attaining significant mAP improvements on M3FD, VEDAI, and LLVIP datasets while adding merely 0.4M parameters [4,26]. In addition, the EEF method introduces an energy score-guided feature enhancement module (EFEM), a lightweight cross-attention fusion module (ECFM), and an adaptive feedback module (AFM), improving fusion quality while reducing computational complexity [27].

### 2.3. Limitations of Existing Methods

In transportation scenarios, single-modality RGB detection can effectively recognize vehicle appearance and color under daytime conditions; however, its performance degrades significantly at night or under strong backlighting. In contrast, IR (IR) detection can stably capture thermal contours of vehicles under low-light or smoky conditions and is particularly advantageous for pedestrian detection [18].

Multispectral fusion methods demonstrate stronger adaptability and robustness in transportation scenarios. For instance, large-scale RGB-IR vehicle detection datasets, such as DroneVehicle, have promoted the development of specialized detection networks. Sun et al. [23] proposed an uncertainty-aware module that assigns weights to detection boxes from different modalities to facilitate training. Shen et al. [20] introduced cross-attention and iterative interaction mechanisms to improve modality alignment and fusion.

The CAGTDet framework combines TSRA and CFT to progressively correct translation, scale, and rotation deviations while enhancing feature complementarity, achieving an mAP of 74.57% on the DroneVehicle dataset and outperforming existing methods [10,14].

In pedestrian detection, multispectral approaches significantly improve recall rates under nighttime and occlusion conditions. Illumination-aware Faster R-CNN enhances fusion robustness by modeling illumination information [18], while MAF-YOLO improves detection accuracy through multimodal attention fusion [5]. EFETN achieves an mAP@50 of 96.4% on the LLVIP nighttime pedestrian detection task, further demonstrating the effectiveness of cross-modal fusion [20,28].

Overall, object detection research in transportation scenarios has evolved from single-modality limitations toward cross-modal complementary fusion. Continuous improvements in feature alignment, fusion strategies, lightweight design, and real-time performance have enabled these methods to better meet the demands of all-weather intelligent transportation systems.

## 3. Materials and Methods

### 3.1. Overall Architecture

**Figure 1:**
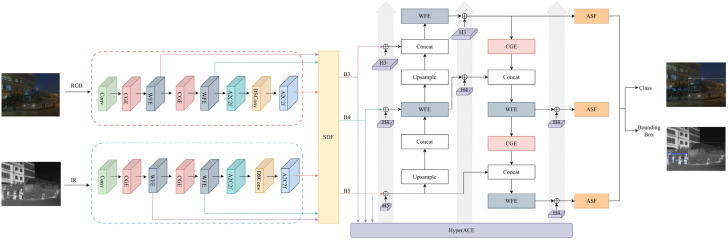
Overall architecture of DMNet.

DMNet is an improved dual-modality object detection network built upon the YOLOv13n architecture, aiming to balance detection performance and computational efficiency. Considering the requirements of real-time deployment and resource-constrained scenarios, YOLOv13n is adopted as the baseline due to its compact structure and relatively low computational cost. The designed framework adopts a dual-stream backbone and is designed from four key aspects, shown in Figure 1: a shallow detail fusion module (SDF), a wavelet-based feature enhancement module (WFE), a global enhancement module (CGE), and an adaptive spatial fusion module (ASF). By progressively refining features at different hierarchical levels, DMNet effectively preserves the complementary advantages of both modalities while improving detection performance under challenging conditions such as complex illumination, background interference, and scale variation.

Specifically, DMNet first employs a dual-stream backbone to encode RGB and IR images in parallel. The RGB branch focuses on extracting texture structures, edge contours, and local appearance features, whereas the IR branch emphasizes thermal radiation responses and salient region distributions of targets. Compared with directly mixing multimodal inputs at the input stage, the dual-stream design avoids early interference between heterogeneous signals, allowing each modality to be fully represented within its own feature space. As the network deepens, multi-scale feature maps are generated from both branches, providing a rich hierarchical basis for subsequent cross-modal interaction.

Preserving small details such as edges, textures, and shape edges is a key objective. We introduce a shallow detail fusion (SDF) module early on, where features first interact. Shallow features possess high spatial resolution and contain abundant fine-grained visual cues, which are crucial for improving small object detection and localization accuracy. However, they are also more susceptible to modality discrepancy and background noise. Direct fusion may cause useful details to be overwhelmed by irrelevant responses. Therefore, SDF first performs feature mapping and alignment across modalities, followed by attention-based adaptive enhancement to highlight discriminative regions while suppressing redundant background responses. As a result, more consistent and refined shallow representations are obtained, providing reliable inputs for subsequent deep semantic modeling.

As features propagate to deeper layers, conventional convolution operations gradually enlarge the receptive field but still struggle to adequately model small objects, weak-texture targets, and objects with significant shape variations in complex environments. To address this limitation, a wavelet-based feature enhancement (WFE) module is introduced to enrich intermediate feature representations. By decomposing features into different frequency components, WFE captures both global structural information and local texture variations. Specifically, low-frequency components preserve object contours and spatial distributions, while high-frequency components enhance edges, textures, and fine details. Through joint reconstruction of multi-frequency information, WFE expands the feature representation capacity without significantly increasing computational cost, enabling the model to better capture subtle variations across multi-scale targets.

As the semantic level increases, feature maps gradually gain stronger semantic abstraction ability, but suffer from detail attenuation and noise accumulation. Particularly in dual-modality fusion scenarios, the absence of global constraints may lead to semantic redundancy or insufficient utilization of complementary information. To address this issue, a global enhancement (CGE) module is designed to recalibrate deep fusion features. By aggregating global contextual information, CGE extracts image-level semantic descriptors and generates channel-wise guidance weights, enabling the network to better distinguish target-related responses from background interference. With global modulation, the discriminability and robustness of deep features are significantly improved, providing more stable semantic support for subsequent prediction.

Since relying on a single-scale feature is insufficient to simultaneously capture fine details of small objects and represent the broader semantics of large ones, an adaptive spatial fusion (ASF) module is further designed in the detection head to integrate complementary information across multiple feature scales. Unlike conventional feature pyramid networks with fixed fusion strategies, ASF dynamically learns spatial weighting based on feature distribution and object location, allowing high-resolution features to better capture small objects and boundary details, while deep features focus on semantic discrimination and global context representation. The resulting multi-scale feature representation effectively balances spatial precision and semantic richness, significantly enhancing the detection capability for dense targets, occluded objects, and targets with large scale variations.

Based on the above design, DMNet performs category prediction and bounding box regression at each detection scale, followed by post-processing to remove redundant detections. Overall, the designed framework establishes a comprehensive optimization pipeline covering shallow detail preservation, cross-modal interaction, deep semantic enhancement, and multi-scale feature fusion, thereby significantly improving the performance of dual-modality object detection in complex environments. The following sections describe the detailed implementation of the SDF, WFE, CGE, and ASF modules.

### 3.2. Surface Detail Fusion (SDF) Module

Aiming to overcome the limitations of traditional pixel-level fusion in RGB–IR dual-modality object detection—namely inaccurate shallow feature alignment, loss of fine-grained details, and background noise interference—we introduce the surface detail fusion (SDF) module, which introduces a joint channel–spatial attention mechanism at the shallow stage of the network. By dynamically generating cross-modal fusion weights, it enables adaptive enhancement and fusion of IR and RGB features, thereby improving shallow feature representation and localization accuracy.

**Figure 2:**
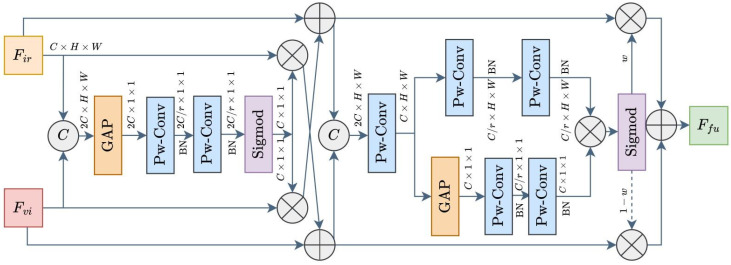
SDF fusion module.

Let the IR and RGB features at the *i* layer be denoted as $F_{ir}^{i}$ and $F_{vi}^{i}$, respectively:

(1) $$\begin{array}{c} F_{ir}^{i},F_{vi}^{i}\in R^{C\times H\times W} \end{array},$$

where *C*, *H*, and *W* represent the number of channels, height, and width of the feature maps, respectively. The SDF module mainly consists of three stages: feature enhancement, fusion weight generation, and feature fusion, as illustrated in Figure 2.

In the feature enhancement stage, the IR and visible features are first concatenated along the channel dimension to obtain a joint feature representation:

(2) $$\;\begin{array}{c} F_{c}=C(F_{ir}^{i},F_{vi}^{i}) \end{array},$$

here, C(⋅) represents the channel concatenation operation. Subsequently, global average pooling (GAP) is performed on the concatenated features, after which a 1 × 1 convolution generates the channel attention weights:

(3) $$\;\begin{array}{c} F_{c}=C(F_{ir}^{i},F_{vi}^{i}) \end{array},$$

(4) $$A_{c}=\sigma \left(Conv_{1\times 1}(GAP(F_{c}))\right),$$

where σ(⋅) denotes the Sigmoid activation function, and the resulting attention weights are used to refine cross-modal feature representation, and the enhanced features are obtained through cross-modal interaction:

(5) $$\;\;\begin{array}{c} {\hat{F}}_{ir}^{i}=F_{ir}^{i}+\left(F_{vi}^{i}⊙A_{c}\right) \end{array},$$

(6) $$\begin{array}{c} {\hat{F}}_{vi}^{i}=F_{vi}^{i}+\left(F_{ir}^{i}⊙A_{c}\right) \end{array},$$

where ⊙ denotes element-wise multiplication. This interaction strategy effectively exploits complementary information between IR and RGB modalities, enabling IR features to enhance thermal responses while preserving structural details in visible features.

In the fusion weight generation stage, the enhanced features are concatenated again to obtain:

(7) $$\begin{array}{c} F_{e}=C({\hat{F}}_{ir}^{i},{\hat{F}}_{vi}^{i}) \end{array},$$

$F_{e}$ is then fed into both the channel attention and spatial attention modules to generate channel weights $A_{C}^{i}$ and spatial weights $A_{S}^{i}$, respectively. The final fusion weight is jointly determined by these two components:

(8) $$\begin{array}{c} W^{i}=\sigma \left(A_{C}^{i}⊙A_{S}^{i}\right) \end{array},$$

where the fusion weight $W^{i}\in [0,1]$ represents the adaptive cross-modal weighting.

In the feature fusion stage, the IR and RGB features are dynamically weighted according to the generated fusion weights to obtain the final fused feature:

(9) $$\begin{array}{c} F_{fu}^{i}=\left(W^{i}⊙{\hat{F}}_{ir}^{i}\right)+\left((1-W^{i})⊙{\hat{F}}_{vi}^{i}\right) \end{array}.$$

As illustrated in Figure 2, the SDF module initially conducts feature enhancement through channel concatenation and attention mechanisms, and then generates fusion weights using joint channel–spatial attention, and finally achieves adaptive fusion via weighted combination of dual-modality features.

Compared with conventional fusion methods, the SDF module exhibits several advantages. First, the cross-modal attention mechanism enhances shallow feature representation and alleviates inconsistencies between RGB and IR features. Second, the integration of channel attention and spatial attention enables spatially adaptive fusion, allowing the model to select the most informative modality in different regions. Third, the module introduces minimal computational overhead by relying only on global average pooling and 1 × 1 convolution operations. Finally, the lightweight design allows flexible integration into shallow or intermediate feature fusion stages, significantly improving dual-modality object detection performance while maintaining computational efficiency.

### 3.3. Wavelet-Based Feature Extraction (WFE)

Conventional convolution operations face limitations in expanding the receptive field—such as parameter redundancy, performance saturation, and insufficient modeling of low-frequency structural information. To alleviate these issues, a wavelet-based feature extraction module, termed WFE, is introduced into the backbone network. Inspired by the core idea of WTConv, this module performs multi-frequency convolution operations in the wavelet domain, enabling small convolution kernels to operate over a larger effective region of the original input. As a result, the receptive field can be effectively expanded while maintaining low parameter complexity, and the model’s ability to capture low-frequency structures and object contours is significantly enhanced. Previous studies have shown that WTConv achieves a substantially larger effective receptive field through cascaded wavelet decomposition and small-kernel depth-wise convolution, with parameter growth only increasing logarithmically with respect to the receptive field.

**Figure 3:**
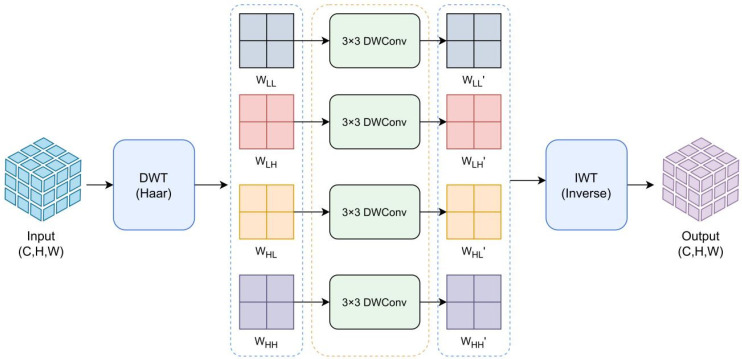
WFE module structure.

Let the input feature map be denoted as:

(10) $$X\in R^{C\times H\times W},$$

where *C*, *H*, and *W* represent the number of channels, height, and width, respectively. The WFE module consists of three main components: wavelet decomposition, frequency-domain convolution, and inverse wavelet reconstruction, as illustrated in Figure 3. In this work, the two-dimensional Haar wavelet is adopted as the transformation basis due to its simplicity, computational efficiency, and orthogonality. Compared with more complex wavelet bases, Haar wavelets can be efficiently implemented using fixed convolutional filters, introducing negligible computational overhead while effectively preserving essential structural and low-frequency information.

For a single-level 2D Haar wavelet decomposition, four sub-band filters can be implemented using depth-wise convolution with stride 2, corresponding to one low-frequency part and three high-frequency part s:

(11) $$\;\;f_{LL}=\frac{1}{2}\left[\begin{array}{cc} 1 & 1\\ 1 & 1 \end{array}\right],\;\;\;\;\;\;\;\;\;\;\;\;\;\;\;\;\;\;\;\;\;\;\;\;\;\;\;\;\;\;\;\;\;\;\;\;\;f_{LH}=\frac{1}{2}\left[\begin{array}{cc} 1 & -1\\ 1 & -1 \end{array}\right],$$

(12) $$f_{HL}=\frac{1}{2}\left[\begin{array}{cc} 1 & 1\\ -1 & -1 \end{array}\right],\;\;\;\;\;\;\;\;\;\;\;\;\;\;\;\;\;\;\;\;\;\;\;\;\;\;\;\;\;\;\;\;\;\;f_{HH}=\frac{1}{2}\left[\begin{array}{cc} 1 & -1\\ -1 & 1 \end{array}\right].$$

Specifically, $f_{LL}$ serves as the low-pass filter for extracting low-frequency approximation information, whereas $f_{LH}$, $f_{HL}$, and $f_{HH}$ are high-pass filters that pick up details along the horizontal, vertical, and diagonal directions, respectively. Applying these filters to the input feature map produces a set of frequency sub-band features:

(13) $$[X_{LL},X_{LH},X_{HL},X_{HH}]\;=Conv([f_{L}L,f_{L}H,f_{H}L,f_{H}H],X),$$

where $X_{LL}$ represents the low-frequency component of the input features, preserving the primary structure and contour information; $X_{LH}$, $X_{HL}$, and $X_{HH}$ denote high-frequency responses in different directions, describing edges, textures, and local details. Since the Haar wavelet filter bank constitutes an orthogonal normalized basis, its inverse transform can be implemented via transposed convolution, thereby reconstructing each frequency component back into the original spatial domain:

(14) $$\begin{array}{c} X=ConvTranspose\left(\left[f_{LL},f_{LH},f_{HL},f_{HH}],[X_{LL},X_{LH},X_{HL},X_{HH}\right]\right) \end{array}.$$

To further expand the receptive field, the WFE module is not limited to single-level wavelet decomposition; instead, it adopts a cascaded decomposition strategy that recursively decomposes only the low-frequency component. In our implementation, two-level wavelet decomposition is adopted to balance representation capability and computational cost. A shallow decomposition is insufficient to effectively enlarge the receptive field, whereas deeper decompositions introduce redundancy and increased complexity. Let $X_{LL}^{\left(i-1\right)}$ denote the low-frequency input at level *i*, then the wavelet decomposition at level i can be expressed as:

(15) $$\begin{array}{c} X_{LL}^{\left(i\right)},X_{LH}^{\left(i\right)},X_{HL}^{\left(i\right)},X_{HH}^{\left(i\right)}=WT(X_{LL}^{\left(i-1\right)}) \end{array},$$

where $X_{LL}^{\left(0\right)}=X$. As the number of decomposition levels increases, the spatial resolution of the low-frequency components decreases progressively while the frequency resolution improves; consequently, subsequent convolution operations can model input information within a larger effective receptive field of the original input. Figure 3 provides an intuitive illustration: when performing a 3 × 3 convolution on the second-level low-frequency component $X_{LL}^{\left(2\right)}$, its actual influence region corresponds to a significantly larger area in the original input, thereby substantially broadening the receptive field.

During the convolution phase, the WFE module first decomposes input features into the frequency domain via wavelet transform, performs deep convolution on each frequency map, and finally reconstructs the output through inverse wavelet transform. For a single-layer WFE, the basic formulation is:

(16) $$\begin{array}{c} Y=IWT(Conv(W,WT(X))) \end{array},$$

where W denotes the parameters of the depth-wise convolution kernel, and WT(⋅) and IWT(⋅) represent the wavelet transform and inverse wavelet transform, respectively. This process not only enables independent convolution on different frequency components but also allows small convolution kernels to model features within a larger region of the original input.

For multi-layer WFE, this paper further employs recursive convolution and progressive reconstruction to aggregate convolution results across different frequencies and scales. Let the set of high-frequency components at level i be formulated as

(17) $$\begin{array}{c} X_{H}^{\left(i\right)}=\{X_{LH}^{\left(i\right)},X_{HL}^{\left(i\right)},\} \end{array}.$$

The frequency-domain convolution at level i can be formulated as:

(18) $$\begin{array}{c} X_{LL}^{\left(i\right)},X_{H}^{\left(i\right)}=WT(X_{LL}^{\left(i-1\right)}) \end{array},$$

(19) $$Y_{LL}^{\left(i\right)},Y_{H}^{\left(i\right)}=Conv(W^{\left(i\right)},(X_{LL}^{\left(i\right)},X_{H}^{\left(i\right)}).$$

Subsequently, leveraging the linearity of the wavelet transform and inverse transform, the outputs from each layer are reconstructed and accumulated progressively. Let $Z^{\left(i\right)}$ represent the aggregated result starting from level *i*; the reconstruction process is given by:

(20) $$\begin{array}{c} Z^{\left(i\right)}=IWT(Y_{LL}^{\left(i\right)}+Z^{\left(i+1\right)},Y_{H}^{\left(i\right)}) \end{array}.$$

Ultimately, the output of the entire WFE module, $Z^{\left(0\right)}$, is obtained. This progressive aggregation mechanism corresponds to the multi-level *WT* → *Conv* → *IWT* pipeline illustrated in Figure 3: shallow high-frequency components primarily retain local edge and texture details, while deeper low-frequency components are responsible for modeling large-scale structural information. The multi-level convolution results are fused step-by-step during the inverse wavelet reconstruction, culminating in output features that integrate both local details and global context.

Functionally, the WFE module offers three advantages over traditional depth-wise convolution. First, by recursively executing convolution on low-frequency components, it enables small-kernel convolutions to operate over a larger original receptive field, thereby significantly enhancing the network’s capability to capture distant cross-modal dependencies. Second, splitting inputs into low and high frequencies lets the model handle overall shape information and fine edge details separately. This multi-frequency response mechanism facilitates the synergistic expression of target structures and details in complex scenarios. Finally, the WFE module serves as a plug-and-play alternative to standard depth-wise convolution layers, requiring no modifications in the overall network architecture and facilitating direct integration into existing CNN detection frameworks. Related studies also indicate that compared to directly increasing the convolution kernel size, WTConv achieves a larger effective receptive field with slower parameter growth and better enhances the response to low-frequency information.

From a complexity perspective, the parameter count of traditional large-kernel convolution typically grows quadratically with kernel size. In contrast, the WFE module converts large-range modeling into small-kernel convolutions across multiple scales via cascaded wavelet decomposition, resulting in logarithmic growth of parameter scale relative to the target receptive field. In other words, for a given requirement of a large receptive field, WFE maintains high modeling capacity without excessive parameterization, which is a key reason why it is well suited as a feature extraction unit in backbone networks.

### 3.4. CGE-Based Context-Guided Downsampling

Preserving critical information during downsampling while enhancing feature representation in complex scenarios is a key challenge. To address this, a context-guided feature enhancement module, termed CGE (context-guided enhancement), is introduced. Drawing inspiration from the context modeling paradigm in CGNet, this module establishes an effective correlation between local features and multi-scale contextual information, which enables effective information enhancement and structural preservation during feature downsampling.

**Figure 4:**
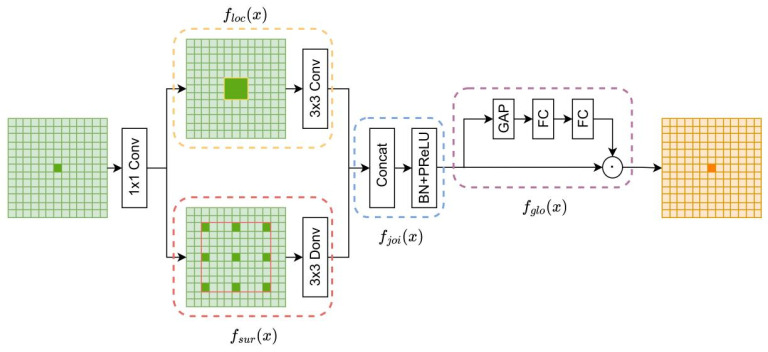
CGE module.

The CGE module is composed of three parts, namely local feature extraction, contextual feature extraction, and feature fusion, as illustrated in Figure 4.

First, in local feature extraction stage, local detail information is extracted via standard convolution, which can be formulated as:

(21) $$\begin{array}{c} F_{loc}=f_{loc}(X) \end{array},$$

where $f_{loc}(\cdot )$ represents the local feature extraction operator, typically implemented by a 3 × 3 standard convolution to capture fine-grained structural information within the neighborhood.

Second, in the contextual feature extraction stage, to enlarge the receptive field and capture distant cross-modal dependencies, dilated convolution is introduced to process the input features:

(22) $$\begin{array}{c} F_{sur}=f_{sur}(X) \end{array},$$

where $f_{sur}(\cdot )$ denotes the contextual feature extraction operator, usually realized by a 3 × 3 convolution. The dilation rate is introduced to efficiently expand the receptive field without increasing kernel size or network depth. This design allows the model to capture broader contextual dependencies, which is particularly important in multimodal scenarios where object appearance may vary significantly across modalities. In our implementation, a moderate dilation rate (e.g., r = 2) is adopted to balance receptive field expansion and computational overhead, avoiding the gridding effect caused by excessively large dilation rates.

Subsequently, the local and contextual features are fused to obtain a joint feature representation:

(23) $$\begin{array}{c} F_{joi}=f_{joi}(F_{loc},F_{sur}) \end{array},$$

where $f_{joi}(\cdot )$ signifies the joint feature extraction operation, generally achieved through channel concatenation followed by a 1 × 1 convolution to integrate multi-scale information.

On this basis, to further incorporate global contextual information, global modeling is performed on the joint features:

(24) $$\begin{array}{c} F_{glo}=f_{glo}(F_{joi}) \end{array},$$

where $f_{glo}(\cdot )$ represents the global feature extraction operator, typically comprising global average pooling (GAP) and point-wise convolution, aimed at capturing global semantic information.

Finally, the input features are fused with the globally enhanced features via a residual connection to yield the output features:

(25) $$\begin{array}{c} Y=X+F_{glo} \end{array}.$$

During the downsampling process, the CGE module reduces spatial resolution by introducing convolution with a stride of 2 or combining pooling operations. Simultaneously, it utilizes the aforementioned context modeling mechanism to compensate for information loss, thereby maintaining feature representation capability while reducing computational load.

Structurally, as depicted in Figure 4, the CGE module enables the network to focus on both fine-grained details and large-scale semantic information through the collaborative modeling of local and contextual features. Compared to traditional downsampling methods, this module offers the following advantages: Firstly, the introduction of dilated convolution effectively expands the receptive field, enhancing the modeling capability for complex scenes. Secondly, the joint feature fusion mechanism achieves efficient integration of multi-scale information. Thirdly, global context guidance improves semantic consistency. Lastly, the incorporation of residual connections helps mitigate gradient vanishing and promotes training stability. Consequently, the CGE module significantly elevates feature representation quality while ensuring computational efficiency.

### 3.5. Adaptive Spatial Feature Fusion (ASF)

**Figure 5:**
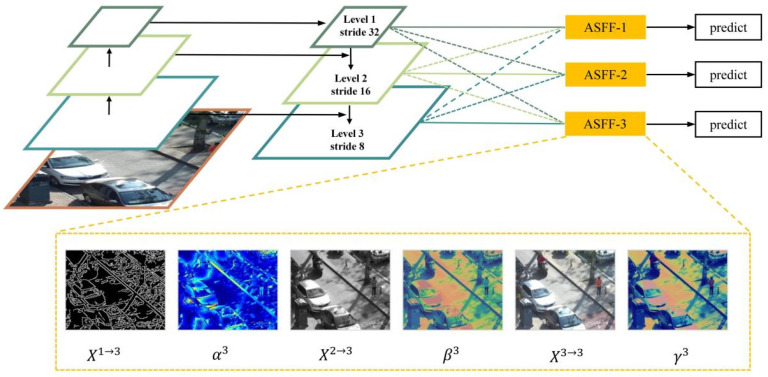
ASF detection head.

In the detection head, we introduce an adaptive spatial feature fusion (ASF) mechanism to address the inherent scale inconsistency issues within the feature pyramid, as illustrated in Figure 5. By learning optimal fusion weights, ASF dynamically integrates features from different levels (P3, P4, and P5). For a specific level l, the fused feature $y_{ij}^{l}$ at spatial location (*i*, *j*) is a weighted sum of rescaled features from all levels:

(26) $$\begin{array}{c} y_{ij}^{l}=\alpha_{ij}^{l}\cdot x_{ij}^{1\to l}+\beta_{ij}^{l}\cdot x_{ij}^{2\to l}+\gamma_{ij}^{l}\cdot x_{ij}^{3\to l} \end{array},$$

the fusion coefficients α,β,γ are constrained by $\alpha_{ij}^{l}+\beta_{ij}^{l}+\gamma_{ij}^{l}=1$ and lie within the range [0, 1], learned via a Softmax-based strategy. This adaptive strategy enables the model to suppress conflicting information from inconsistent scales while amplifying the most representative features for each detection target.

### 3.6. Loss Function Design

In object detection, the training objective aims to minimize the discrepancy between prediction results and ground-truth annotations. Addressing challenges such as significant scale variation, blurred boundaries, and imbalanced sample distribution in complex scenes, we adopt a joint loss function comprising classification, bounding box regression, and distribution focal losses (DFL). The overall formulation is:

(27) $$\begin{array}{c} L_{total}=\lambda_{cls}L_{cls}+\lambda_{box}L_{box}+\lambda_{dfl}L_{dfl} \end{array},$$

where $\lambda_{cls}$, $\lambda_{box}$, and $\lambda_{dfl}$ are the weighting coefficients for the classification loss, localization loss, and distribution focal loss, respectively.

(1) Classification Loss Function

Traditional object detection methods typically employ hard labels based on One-Hot encoding for supervision, which fails to adequately consider localization quality and easily leads to inconsistencies between classification confidence and localization accuracy. To this end, this paper introduces the task-aligned assigner strategy, which generates continuous soft labels as supervision signals by fusing classification scores and localization quality (*IoU*) information.

Based on this, binary cross entropy (BCE) loss is utilized to optimize the classification branch, expressed as:

(28) $$\begin{array}{c} L_{cls}=-\frac{1}{N_{pos}}\sum_{i\in pos}\left[t_{i}\log(\sigma (x_{i}))+(1-t_{i})\log(1-\sigma (x_{i}))\right] \end{array},$$

where $x_{i}$ represents the classification logits output by the network, σ(⋅) denotes the Sigmoid activation function, $t_{i}\in [0,1]$ is the target score generated by the task-aligned strategy, and $N_{pos}$ is the number of positive samples.

Furthermore, analyzing the gradient of the BCE loss with respect to the predicted value yields:

(29) $$\begin{array}{c} \frac{\partial L_{cls}}{\partial x_{i}}=\sigma (x_{i})-t_{i} \end{array}.$$

This demonstrates that the optimization direction of the classification branch depends not only on the prediction error but is also modulated by the quality of the target score. In other words, the model ends up paying more attention to high-quality positive samples.

(2) Bounding Box Regression Loss Function

In bounding box regression tasks, traditional *IoU* loss only considers overlap area and is prone to gradient vanishing when no overlap exists between the predicted box and the ground-truth box. Therefore, we use complete intersection over union (*CIoU*) as the regression loss, introducing constraints on center point distance and aspect ratio in addition to *IoU*.

*CIoU* is defined as:

(30) $$\begin{array}{c} CIoU=IoU-\frac{\rho^{2}(b,b^{gt})}{c^{2}}-\alpha v \end{array},$$

where $\rho (b,b^{gt})$ is the Euclidean distance between the centers of the predicted bounding box and the ground-truth box, *c* is the length of the diagonal of the smallest rectangle that can enclose both boxes, *v* measures the aspect ratio consistency of the two boxes, and α is a coefficient used to balance the contribution of this term in the overall loss.

Consequently, the bounding box regression loss can be expressed as:

(31) $$\begin{array}{c} L_{box}=\frac{1}{N_{pos}}\sum_{i\in pos}w_{i}\left(1-CIoU(B_{i}^{pred},B_{i}^{gt})\right) \end{array},$$

where $w_{i}$ represents the weight generated based on the target score.

Additionally, in oriented object detection tasks, to address the angle periodicity problem, probabilistic *IoU* (ProbIoU) is adopted as a supplement, formulated as:

(32) $$\begin{array}{c} L_{obb}=1-\exp\left(-\frac{1}{2}BD\right) \end{array},$$

where *BD* represents the Bhattacharyya distance between the Gaussian distributions, one built from the predicted box, the other from the ground-truth box.

(3) Distribution Focal Loss Function

In complex scenarios, object boundaries often exhibit uncertainty, while traditional regression methods treat coordinates as deterministic values, struggling to accurately describe the distribution characteristics of real boundaries. Hence, we introduce distribution focal loss (DFL) to model bounding box regression as a discrete probability distribution problem.

A continuous ground-truth coordinate y can be represented by two adjacent discrete points:

(33) $$\begin{array}{c} y=(1-\delta )y_{i}+\delta y_{i+1} \end{array},$$

where *δ* is the interpolation weight. Let the network predict the probabilities for the corresponding discrete positions as $P_{i}$ and $P_{i+1}$, then DFL is expressed as:

(34) $$\begin{array}{c} L_{dfl}=-\left[(y_{i+1}-y)\log P_{i}+(y-y_{i})\log P_{i+1}\right] \end{array}$$

By modeling probability distributions, this method enables the model to learn finer spatial structural information, thereby enhancing boundary localization accuracy.

## 4. Results

### 4.1. Datasets and Evaluation Metrics

#### 4.1.1. Dual-Modal Image Object Detection Dataset

To evaluate the performance of DMNet in a comprehensive manner, experiments are conducted on three representative dual-modal object detection datasets, including M3FD, LLVIP, and VEDAI. These datasets cover diverse scenarios such as urban traffic, nighttime environments, and aerial imagery, providing a reliable benchmark for assessing the effectiveness and generalization capability of dual-modality detection models.

The M3FD dataset [29] contains paired RGB and IR images captured under diverse conditions, like daytime, nighttime, and adverse weather scenarios. It includes multiple object categories such as pedestrians, vehicles, and cyclists, making it suitable for evaluating multi-class detection performance in complex environments.

The LLVIP dataset [6] focuses on low-light conditions and provides high-quality paired RGB–IR images. It is specifically designed for pedestrian detection in nighttime scenarios and has been widely used to evaluate the robustness of multispectral detection methods under poor illumination conditions.

The VEDAI dataset [30] is a benchmark dataset for vehicle detection from an aerial remote sensing perspective, captured by RGB and IR aerial cameras. In the experiments, approximately 1050 pairs of images were used, from which 512 × 512 patches were cropped to form the training and validation sets. It is mainly used for tiny detection.

#### 4.1.2. Evaluation Metrics

Multiple evaluation metrics from the COCO protocol are adopted to assess the designed model, including mean average precision (mAP), precision (P), recall (R), and efficiency-related metrics.

mAP is the go-to evaluation metric for object detection, measuring the overall detection performance across different categories. In this work, two forms of mAP are employed, namely mAP@0.5 and mAP@0.5:0.95. The former uses an *IoU* threshold of 0.5. The latter one averages mAP over *IoU* thresholds from 0.5 to 0.95 in steps of 0.05. Compared to a single threshold, mAP@0.5:0.95 gives a more complete picture of how well the model handles different localization precision requirements. This metric is also the standard in COCO.

We also look at precision and recall to get a better sense of detection quality. Precision is the fraction of correct detections among all predictions:

(35) $$Precision=\frac{TP}{TP+FP},$$

Recall is the fraction of ground-truth objects that get detected:

(36) $$Recall=\frac{TP}{TP+FN},$$

where TP is true positives, FP is false positives, and FN is false negatives. Higher precision means fewer false alarms; higher recall means fewer missed detections.

Above accuracy, we care about efficiency. We use parameter count (Params) and floating-point operations (GFLOPs) to measure computational cost, and inference time per image to gauge real-time performance. Together, these give a balanced view of the model’s accuracy and efficiency.

### 4.2. Implementation Details

Our implementation is built on Ultralytics YOLO (v8.3.13). The training setup uses Python 3.12.3 and PyTorch 2.8.0 with CUDA 12.8. All experiments run on a server with an NVIDIA GeForce RTX 5090 GPU (32 GB memory).

We set input resolution to 512 × 512 and batch size to 8. Training goes for 200 epochs, and we use stochastic gradient descent (SGD) [31] as the optimizer.

To avoid overfitting and accelerate training, early stopping is applied: training is terminated if the validation performance does not improve for 50 consecutive epochs. Automatic mixed precision (AMP) is enabled to improve computational efficiency, and the number of data loading workers is set to 8 to ensure efficient data loading.

For data augmentation, we keep Mosaic turned on throughout. It helps the model generalize better in complex scenes. All experiments use the same training strategy, dataset splits, and hyperparameters to keep comparisons fair.

### 4.3. Ablation Experiments

Ablation experiments are designed on the M3FD dataset to verify the contribution of each designed module. The complete model serves as the baseline, and each of the four core modules—SDF, WFE, CGE, and ASF—is removed individually to analyze their impact on detection accuracy and computational complexity. The results are summarized in Table 1.

Drop the SDF module and performance takes a clear hit. mAP@0.5 goes from 78.4% down to 75.8%, and mAP@0.5:0.95 drops from 53.7% to 51.6%. This tells us that SDF helps with cross-modal feature interaction and fine-grained fusion between RGB and IR. Sure, removing it slightly reduces computation, but the accuracy loss is obvious.

Without WFE, performance declines to 76.9% mAP@0.5 and 52.3% mAP@0.5:0.95. This confirms that the WFE module, through wavelet-based frequency-domain feature extraction, enhances the model’s ability to pick up multi-scale structural details in complex scenes.

Removing the CGE module reduces mAP@0.5 from 78.4% to 77.2%. Meanwhile, computational cost increases, with FLOPs rising from 14.0 G to 15.28 G, parameters from 5.72 M to 6.55 M, and inference time from 5.6 ms to 8.7 ms. This indicates that CGE enhances feature representation efficiency through more effective contextual modeling.

The most severe performance degradation occurs when the ASF detection head is removed: mAP@0.5 drops to 74.7% and mAP@0.5:0.95 to 50.3%. This demonstrates that ASF effectively alleviates scale inconsistencies across feature pyramids and is critical in detecting objects at different sizes.

Overall, all modules contribute positively to the model performance. Among them, SDF and ASF have the most significant impact on detection accuracy, while WFE and CGE mainly enhance feature representation and contextual modeling capability. These results validate the effectiveness of the designed multimodule fusion framework.

### 4.4. Comparative Experiments

To further demonstrate the effectiveness of DMNet, we carry out comprehensive comparisons with several mainstream multimodal object detection methods. All models are trained and evaluated under identical experimental settings for unbiased comparison. The compared methods include several representative state-of-the-art cross-modal fusion approaches, such as MFM-YOLO [32], ICANet [33], DFANet [34], IRMBNet [35], MBNet [36], and more recent methods including EEF [27], CFT [37], CAGTDet [10,14], and EFETN [28].

Table 2 presents a comprehensive comparison of different multimodal object detection methods on the M3FD dataset. The designed DMNet achieves the best performance across all evaluation metrics, with an mAP@0.5 of 78.4% and mAP@0.5:0.95 of 53.7%, significantly outperforming all competing methods. Compared with the strongest baseline CAGTDet, which achieves 73.2% mAP@0.5 and 49.1% mAP@0.5:0.95, our method improves performance by 5.2% and 4.6%, respectively. Similarly, DMNet surpasses MFM-YOLO (72.8%, 48.6%) and ICANet (72.6%, 48.6%) by clear margins. In terms of detection accuracy, DMNet achieves the highest precision of 86.2% and recall of 71.8%, exceeding all compared methods. Further, the model maintains a moderate complexity with 5.72M parameters and 14.0 GFLOPs, demonstrating a favorable balance between performance and efficiency compared to lightweight baselines such as MBNet and EEF.

As shown in Table 3, a detailed per-category analysis further validates the effectiveness of DMNet across different object classes. In large-object categories such as Car and Bus, DMNet achieves 90.0% and 90.2%, respectively, outperforming all competing methods. For the Truck category, our model reaches 81.4%, consistently higher than other approaches.

In more challenging small-object scenarios, DMNet achieves 66.2% on Motorcycle, significantly surpassing representative methods such as CAGTDet (57.5%), EFETN (56.8%), and MFM-YOLO (57.0%). In the Lamp category, DMNet achieves 62.5%, which is also the highest among all compared methods. For the People category, DMNet attains 80.1%, maintaining superior performance over all baselines. Overall, DMNet achieves the best average performance of 78.4%, demonstrating consistent advantages across all categories.

**Figure 6:**
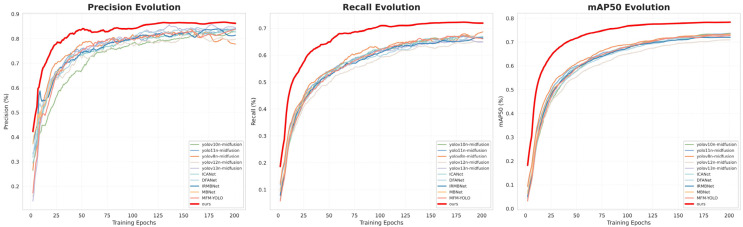
Training curve comparison of precision, recall and mAP among different models.

To further evaluate training behavior, precision, recall, and mAP@0.5 curves during training are illustrated in Figure 6. The designed method achieves rapid performance improvement in the early stages and maintains stable convergence in later stages. Compared with other methods, it consistently achieves higher precision, recall, and mAP values. In addition, the training curves are smoother and more stable, indicating better training stability and generalization capability.

**Figure 7:**
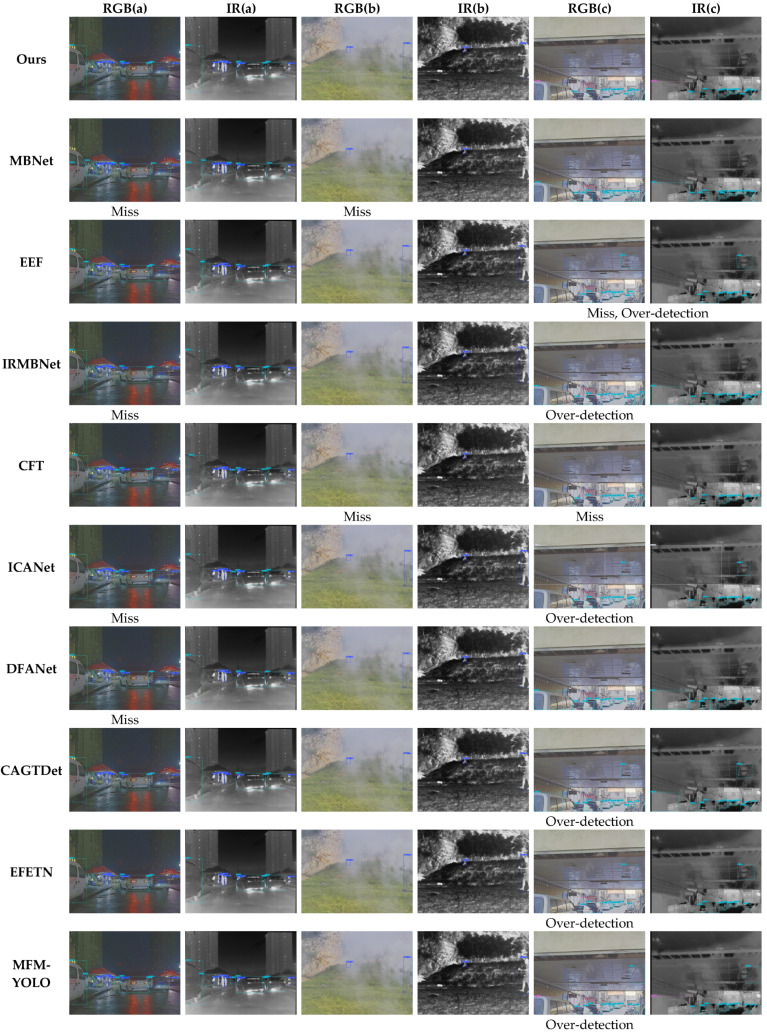
Detection results visualization on the M3FDdataset.

To qualitatively assess the robustness of DMNet in complex real-world multimodal environments, Figure 7 compares our approach with several representative baselines. The annotations beneath each subfigure highlight typical failure cases of baseline models under challenging conditions like low illumination, fog interference, and complex background transitions. These failures mainly include missed detections and over-detection.

These qualitative results clearly indicate that DMNet delivers more robust detection performance across diverse scenarios by effectively leveraging the complementary strengths of RGB and IR modalities, thereby reducing common errors observed in state-of-the-art methods and achieving more accurate and stable detection outcomes.

### 4.5. Generalization Experiments

To evaluate the robustness of DMNet across different scenarios, we conduct additional robustness evaluation on two multimodal datasets, LLVIP and VEDAI, which represent distinct application settings with variations in illumination, viewpoint, target scale, and background complexity. All experiments on LLVIP, VEDAI, and M3FD are conducted independently using identical training settings. The results demonstrate that DMNet maintains stable and consistent performance across datasets with different characteristics.

As shown in Table 4, DMNet still maintained outstanding performance on datasets with different characteristics:

On the LLVIP dataset, DMNet achieves 94.4% mAP@0.5 and 58.2% mAP@0.5:0.95, with precision and recall reaching 93.6% and 89.2%, respectively. These results demonstrate the robustness of DMNet under nighttime low-light surveillance conditions.

On the VEDAI dataset, in the face of complex ground backgrounds and tiny vehicle targets, the overall mAP@0.5 of the model reached 59.0%, and the mAP@0.5:0.95 reached 34.5%. Accuracy for categories like “Pickup” (67.9%), “Tractors” (63.6%), and “Camping cars” (57.4%) remains high, and even for challenging small targets such as “Van” (44.0%) and “Truck” (39.7%), the model retains robust performance, highlighting its ability to capture multi-scale targets.

Inference time in Table 5 shows that DMNet requires only 4.0 ms (LLVIP) and 8.0 ms (VEDAI), validating the practical efficiency of the model under different computational loads. The designed model proves capable in adapting to varied data domains without compromising its lightweight design.

To intuitively demonstrate the generalization capability of DMNet across multimodal and cross-domain scenarios, we provide qualitative visualization results.

**Figure 8:**
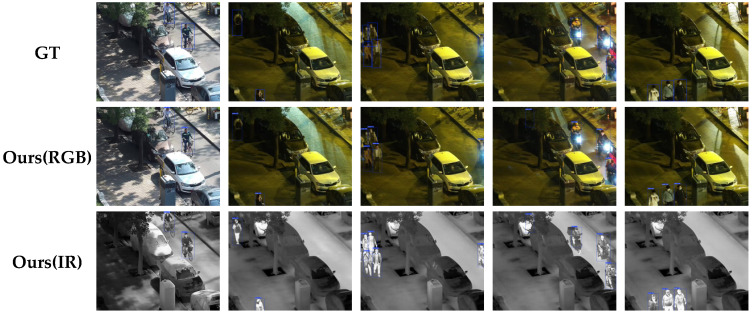
Detection results visualization on the LLVIP dataset.

Figure 8 illustrates the results on the LLVIP dataset. In scenarios where visible images suffer from severe darkness and pedestrians are barely discernible, the IR modality preserves clear thermal signatures. By leveraging the adaptive cross-modal fusion mechanism, DMNet successfully restores targets obscured by darkness and generates bounding boxes that align closely with the ground-truth annotations.

**Figure 9:**
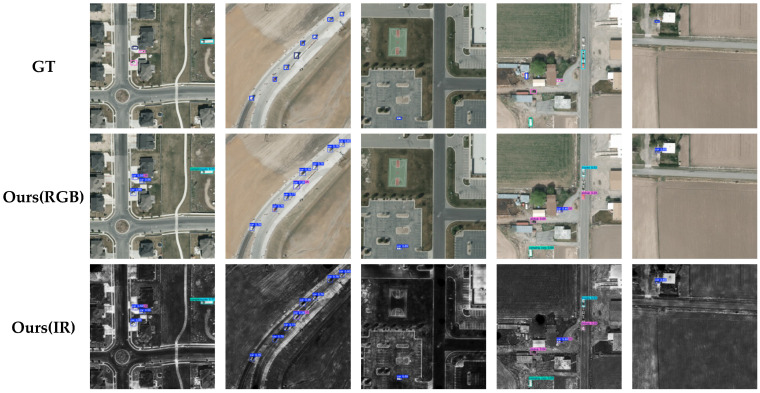
Detection results visualization on the VEDAI dataset.

Figure 9 presents the detection results on the VEDAI dataset. Faced with complex textured backgrounds and densely distributed small-scale vehicles, DMNet effectively suppresses background clutter while accurately localizing tiny targets. Even when targets are extremely small and exhibit low contrast against the background, the model maintains high recall and localization accuracy.

Overall, the designed method achieves stable and competitive performance across different datasets and scenarios, demonstrating robust and consistent performance across diverse scenarios.

## 5. Discussion

Although DMNet demonstrates superior performance across multiple dual-modality object detection benchmarks, especially in complex scenarios, with small object, and low-light conditions, several critical issues remain that warrant further investigation from both practical deployment and methodological perspectives.

Most existing dual-modality detection studies, including those based on the M3FD, LLVIP, and VEDAI datasets adopted in this work, rely on the ideal assumption that RGB and IR images are strictly aligned at the pixel level. However, in real-world applications, cross-modal inputs are often affected by sensor baseline offsets, mechanical vibrations, temporal synchronization errors, and calibration drift. These factors lead to spatial misalignment and boundary inconsistency, which become more pronounced in scenarios involving fast motion or large parallax.

The SDF module introduces a channel–spatial dual-attention mechanism to enhance cross-modal alignment and detail representation at the shallow feature level. Experimental results indicate that this module improves small-object localization accuracy and boundary clarity. Nevertheless, when the degree of physical misalignment exceeds the modeling capacity of the network, especially under severe parallax or motion blur conditions, the effectiveness of SDF may be constrained. In such cases, the model may incorrectly treat RGB and IR features from the same object as separate instances, thereby reducing detection accuracy and stability. Similarly, although the WFE module expands the receptive field and enhances multi-scale representation through frequency-domain decomposition, its implicit reliance on input alignment and geometric consistency is not fully eliminated. Therefore, improving robustness under weakly aligned or unaligned conditions remains a key challenge for practical multimodal perception systems.

The WFE module enhances multi-scale structural modeling by decomposing features into frequency components while maintaining low computational overhead. When we removed it, mAP@0.5 dropped by 1.5%, confirming its contribution to capturing structural information in complex environments.

The CGE module fixes the context loss from downsampling by stitching local and global features via skip connections and multi-scale fusion. Although its removal has a relatively minor impact on accuracy, it significantly increases computational complexity and inference latency, indicating a trade-off between accuracy and speed in practical deployment. These observations suggest that while frequency-domain modeling and contextual enhancement are theoretically beneficial, their practical effectiveness depends on further optimization of computational efficiency.

Among all components, the ASF module has the most significant impact on detection performance, particularly for multi-scale and small-object scenarios. Ablation results indicate that removing ASF results in a 3.7% decrease in mAP@0.5, highlighting its critical role. This finding suggests that feature inconsistency across pyramid levels is a major challenge in multimodal detection. By dynamically learning spatial weights, ASF effectively mitigates scale conflicts and enhances feature integration. Combined with SDF, the designed framework achieves improved small-object detection performance while maintaining competitive inference speed.

In our future work, we will try to loosen the strict alignment assumption and improve model robustness under weakly aligned or unaligned conditions. For instance, deformable cross-attention mechanisms could be introduced to enable adaptive cross-modal interaction. In addition, the lightweight design of DMNet makes it suitable for resource-limited setups like UAVs, mobile robots, and embedded IoT systems. Further optimization of the SDF and ASF modules may facilitate real-time applications in intelligent surveillance, disaster response, and nighttime monitoring. Moreover, integrating multimodal perception with vision–language models and reinforcement learning may enable advanced applications such as autonomous navigation and decision-making in complex environments.

## 6. Conclusions

We designed an RGB–IR dual-modality object detection framework, termed DMNet, to address the challenges of illumination variations, background interference, and multi-scale object detection in complex environments. The architecture integrates four core components: the surface detail fusion (SDF) module for shallow feature alignment, the wavelet feature extraction (WFE) module for frequency-domain enhancement, the context-guided enhancement (CGE) module for semantic refinement, and the adaptive spatial fusion (ASF) head for multi-scale feature aggregation.

Extensive experiments on three challenging multimodal datasets, namely VEDAI, M3FD, and LLVIP, demonstrate that DMNet consistently outperforms existing methods. With only 5.72M parameters and 14.0 GFLOPs, the designed model achieves 78.4% mAP@0.5 and 53.7% mAP@0.5:0.95 on M3FD, and 94.4% mAP@0.5 on LLVIP. These results indicate that DMNet effectively balances detection accuracy, computational efficiency, and robustness, making it suitable for deployment in real-world scenarios.

To summarize, we developed an effective and efficient solution for multimodal object detection in complex environments. Future work will extend into improving robustness under non-ideal data conditions and exploring broader applications in multimodal perception and intelligent systems.

## Figures and Tables

**Table 1:** Ablation study of different modules on the M3FD dataset.

Number	SDF	WFE	CGE	ASF	mAP@0.5 (%)	mAP@0.5:0.95 (%)	Parameters (M) ↓	FLOPs (G) ↓	Inference Time (ms) ↓
1	√	√	√	√	**78.4**	**53.7**	5.72	14	5.6
2	×	√	√	√	75.8	51.6	5.22	13.2	**4.6**
3	√	×	√	√	76.9	52.3	5.78	14.2	8.4
4	√	√	×	√	77.2	51.4	6.55	15.28	8.7
5	√	√	√	×	74.7	50.3	**4.02**	**10.6**	9.8

Note: Bold numbers indicate the best performance achieved among all compared methods for the corresponding metric. √ indicates the module is adopted, while × indicates it is not. Arrows (↓) denote that lower values are preferable for the corresponding metric.

**Table 2:** Comparison of different multimodal object detection networks.

Method	mAP@0.5	mAP@0.5:0.95	P	R	Parameters (M) ↓	FLOPs (G) ↓	Inference Time (ms) ↓
Ours	**78.40**	**53.70**	**86.2**	**71.8**	5.72	14.0	5.6
MBNet [36]	53.5	31.8	70.4	48.9	**2.48**	**8.3**	**2.5**
EEF [27]	71.5	47.2	81.5	64.8	3.95	10.8	3.6
IRMBNet [35]	72.2	47.9	84	65.4	4.09	11.6	3.7
CFT [38]	71.8	47.5	82.1	65.0	3.70	10.5	3.4
ICANet [33]	72.6	48.6	82.6	66.3	6.9	11.2	15.6
DFANet [37]	72.7	48.5	82	67.3	4.14	11.7	5.9
CAGTDet [10,14]	73.2	49.1	83.5	67.8	5.10	12.5	5.2
EFETN [28]	72.9	48.8	83.0	66.9	4.50	12.0	4.6
MFM-YOLO [32]	72.8	48.6	84.3	65.8	3.58	9.7	3.8

Note: Bold numbers indicate the best performance achieved among all compared methods for the corresponding metric.

**Table 3:** Detection performance comparison of different multimodal object detection networks for each category.

Method	Car	Bus	Truck	Motorcycle	Lamp	People	Average
Ours	**90.0**	**90.2**	**81.4**	**66.2**	**62.5**	**80.1**	**78.4**
MBNet	76.7	67.1	51.9	29.7	33.2	62.1	53.5
EEF	86.5	86.2	75.6	55.8	54.6	73.7	71.5
IRMBNet	86.9	86.8	75.9	54.3	55.3	74.0	72.2
CFT	86.7	86.5	75.8	55.9	54.8	74.0	71.8
ICANet	87.3	86.9	76.2	56.4	54.0	74.8	72.6
DFANet	86.8	87.3	77.8	56.3	53.8	74.2	72.7
CAGTDet	87.6	87.4	77.0	57.5	55.8	75.6	73.2
EFETN	87.1	87.0	76.5	56.8	55.2	74.9	72.9
MFM-YOLO	87.3	86.4	76.0	57.0	54.9	75.1	72.8

Note: Bold numbers indicate the best performance achieved among all compared methods for the corresponding metric.

**Table 4:** Performance of DMNet onLLVIP and VEDAI dataset under independent training.

Datasets	Model	Test IMG	Test Instance	mAP@0.5	mAP@0.5:0.95	P	R	Inference Time (ms)
LLVIP	Ours	3463	7931	94.4	58.2	93.6	89.2	4.0
VEDAI	Ours	308	814	59.0	34.5	59	60.3	8
M3FD	Ours	840	6904	78.4	53.7	86.2	71.8	5.6

**Table 5:** Category-level detection performance on LLVIP and VEDAI datasets.

Dataset	Category	mAP@0.5	mAP@0.5:0.95	P	R
LLVIP	all (person)	94.4	58.2	93.6	89.2
VEDAI	car	81.3	47.8	75.6	72.6
	pickup	67.9	42.4	54.7	80.8
	tractors	63.6	29.6	74.0	57.4
	camping cars	57.4	35.2	53.1	68.3
	vans	44.0	27.7	51.5	36.6
	trucks	39.7	24.1	45.1	45.8
	all	59	34.5	59	60.3

## Data Availability

Publicly available datasets were analyzed in this study. These datasets can be found here: the VEDAI dataset (https://downloads.greyc.fr/vedai/, accessed on 3 January 2026), the M3FD dataset (https://github.com/JinyuanLiu-CV/TarDAL, accessed on 3 January 2026), and the LLVIP dataset (https://bupt-ai-cz.github.io/LLVIP/, accessed on 3 January 2026).
